# Longitudinal stability in working memory and frontal activity in relation to general brain maintenance

**DOI:** 10.1038/s41598-022-25503-9

**Published:** 2022-12-05

**Authors:** Lars Nyberg, Nina Karalija, Goran Papenberg, Alireza Salami, Micael Andersson, Robin Pedersen, Tomas Vikner, Douglas D. Garrett, Katrine Riklund, Anders Wåhlin, Martin Lövdén, Ulman Lindenberger, Lars Bäckman

**Affiliations:** 1 Department of Radiation Sciences, Umeå University, 90187 Umeå, Sweden; 2 Department of Integrative Medical Biology, Umeå University, 90187 Umeå, Sweden; 3 Umeå Center for Functional Brain Imaging (UFBI), Umeå University, Umeå, Sweden; 4 Aging Research Center, Karolinska Institutet & Stockholm University, Tomtebodavägen 18A, 17165 Stockholm, Sweden; 5 Center for Lifespan Psychology, Max Planck Institute for Human Development, Lentzeallee 94, 14195 Berlin, Germany; 6 Max Planck, UCL Centre for Computational Psychiatry and Ageing Research, Lentzeallee 94, 14195 Berlin, Germany; 7 Department of Applied Physics and Electronics, Umeå University, 90187 Umeå, Sweden; 8 Department of Psychology, University of Gothenburg, Gothenburg, Sweden

**Keywords:** Cognitive control, Cognitive ageing

## Abstract

Cognitive functions are well-preserved for some older individuals, but the underlying brain mechanisms remain disputed. Here, 5-year longitudinal 3-back in-scanner and offline data classified individuals in a healthy older sample (baseline age = 64–68 years) into having stable or declining working-memory (WM). Consistent with a vital role of the prefrontal cortex (PFC), WM stability or decline was related to maintained or reduced longitudinal PFC functional responses. Subsequent analyses of imaging markers of *general* brain maintenance revealed higher levels in the stable WM group on measures of neurotransmission and vascular health. Also, categorical and continuous analyses showed that rate of WM decline was related to global (ventricles) and local (hippocampus) measures of neuronal integrity. Thus, our findings support a role of the PFC as well as general brain maintenance in explaining heterogeneity in longitudinal WM trajectories in aging.

## Introduction

Cognitive functions are vital for many forms of human behavior. Age-related cognitive decline is normative and can have profound implications for the affected individual as well as the society. Therefore, a main focus of contemporary research concerns how to explain well-preserved cognition in some older adults^1^, as insights into this issue may contribute to successful intervention and prevention. One influential perspective is that of *brain maintenance*^2^, which holds that older adults with well-preserved cognition have maintained a ‘youth-like’ brain. Support for brain maintenance comes from studies linking specific cognitive functions to certain aspects of brain integrity, such as episodic memory with maintained medial-temporal lobe (MTL) structure and function^3–5^ and working memory (WM) with a youth-like or stable activity pattern in prefrontal cortex (PFC) regions^6,7^.

Intriguingly, recent behavioral findings show a strong dependency between changes in fluid and crystallized abilities in aging^8^. Relatedly, well-preserved episodic memory in aging has been shown to generalize to executive function and processing speed^5,9^. Such commonalities direct attention toward domain-general drivers of maintenance, and the possibility of *general* brain maintenance has been proposed^10^. Generality implies that a certain form of brain and cognitive maintenance generalizes to other kinds of maintenance. Testing the generality of maintenance requires longitudinal data on multiple markers of cognition and brain integrity.

Here, we analyzed 5-year longitudinal data from 124 older participants in the Cognition, Brain, and Aging (COBRA) study^11,12^. A numerical 3-back WM task was administered in the magnetic resonance imaging (MRI) scanner as well as during offline testing, and stability or decline was *jointly* determined by in-scanner and offline performance on this demanding WM task. The stable and declining groups were also compared on offline measures of crystallized (vocabulary) and fluid (processing speed) abilities, which in our previous cross-sectional analyses were related to WM performance^13^.

Next, WM behavior was related to PFC functional responses. At baseline, given that the groups would have comparable WM performance we predicted comparable levels of brain activity, alternatively higher activity in the decline group reflecting compensation for emerging change^14,15^. In the longitudinal analyses, based on meta-analytic evidence of robust age differences in PFC activity during *n*-back task performance^16^, we predicted that individuals with longitudinal decline would show reduced PFC activity over time whereas stable WM performance would relate to maintained PFC function^7^.

Finally, we explored MRI- and positron emission tomography (PET) imaging markers of three putative underlying mechanisms of general brain maintenance^10^: (i) neuronal integrity, (ii) neurotransmission, and (iii) vascular health. Global (ventricular size) and local (hippocampus) MRI-measurements of atrophy were used as in vivo markers of neuronal integrity^17^. For neurotransmission, we analyzed dopamine-system integrity using a PET-measure of striatal dopamine D2 receptor binding. Dopamine has been suggested to have a key role in cognitive aging^18^, and we expected a relation between D2 binding and better maintained WM. For cerebrovascular health we analyzed white-matter lesion load and arterial pulsatility, which is a novel MRI marker sensitive to age-related vascular stiffness and resistance^19^. Finally, we compared the groups on physical activity levels, as this lifestyle factor has been related to brain maintenance^20^.

## Results

**Figure 1:**
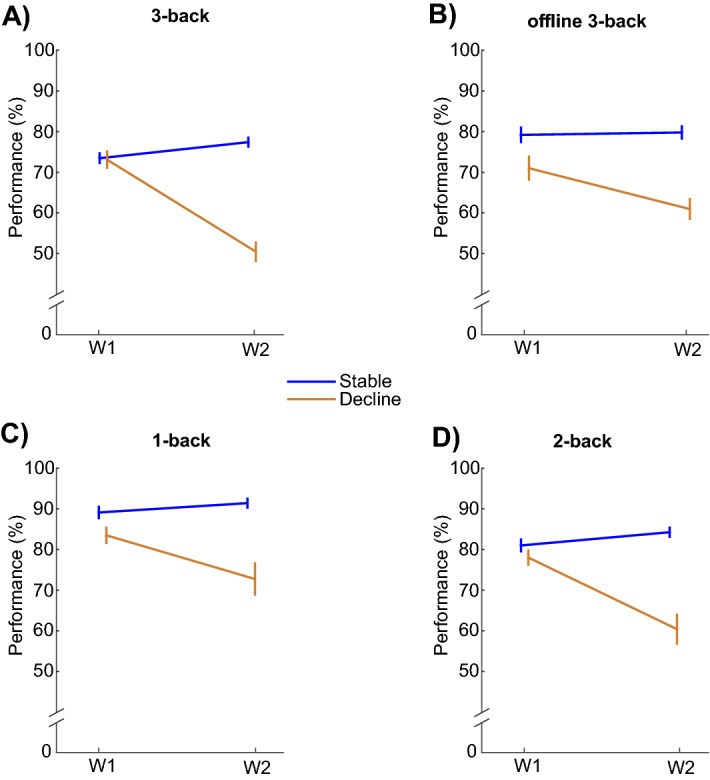
Heterogeneity in WM trajectories. Longitudinal 3-back trajectories (**A**,**B**) classified individuals into stable versus declining WM. The distinction generalized to lower levels of WM load (**C**,**D**). (Mean ± 1SE). W = Wave.

Longitudinal data on the in-scanner 3-back task identified 68 individuals with stable WM and 35 with declining WM (the latter had above-chance performance at baseline followed by reduction at follow-up; see “Methods” and Fig. 1A). Corresponding analyses of the offline 3-back task revealed that 55 were stable also on the offline task, and 25 showed decline also on the offline task. These 80 individuals, with converging data on stability or decline from the in-scanner and offline 3-back tasks, constituted the main sample in analyses of general brain maintenance (see Supplementary Fig. S1). The declining group was slightly older [M_Stable_ = 65.9 y, M_Decline_ = 66.6 y; t(78) = 2.3, p = 0.024] and less educated [M_Stable_ = 14.2 y, M_Decline_ = 12.6 y; t(78) = 2.1, p = 0.036] than the stable group. There was no group difference in proportion of ApoE ε4 carriers (ε4_Stable_ = 26%, (ε4_Decline_ = 28%) or gender distribution (% female/male Stable = 42/58; Decline = 48/52). Figure 1 shows longitudinal in-scanner (A) and offline (B) 3-back performance for the main sample. The data patterns for 1-back and 2-back (Fig. 1C,D) mimicked those for 3-back.

### Behavioral analyses of fluid and crystallized abilities

Analyses of group differences in fluid and crystallized ability revealed faster processing speed at both waves in the stable group, [Stable wave 1: M = 53.2, SD = 8.7; Stable wave 2: M = 53.4, SD = 8.4, Decline wave 1: M = 47.7, SD = 8.4; Decline wave 2: M = 47.3, SD = 9.1: main effect of group: F(1,75) = 8.1, p = 0.006]. A similar pattern was seen for vocabulary, [Stable wave 1: M = 24.2, SD = 3.0; Stable wave 2: M = 24.4, SD = 2.7, Decline wave 1: M = 22.8, SD = 3.0; Decline wave 2: M = 22.5, SD = 2.5: main effect of group: F(1,76) = 6.2, p = 0.015].

### Functional brain activity in relation to WM stability and decline

**Figure 2:**
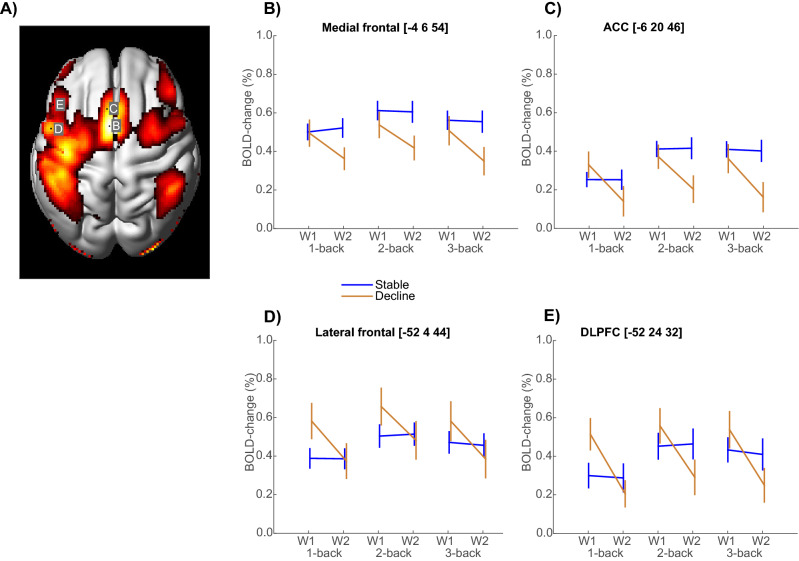
Functional brain activity during *n*-back tests of WM. (**A**) Brain regions showing differential BOLD signal during *n*-back in stable and declining persons (pFWE < 0.05). (**B**–**E**) Significant group differences in PFC longitudinal trajectories. (Mean ± 1SE). W = Wave.

To identify regions in which the BOLD signal was altered by WM demands, the three WM-conditions (1/2/3-back) were contrasted with fixation at the first wave for participants with above-chance performance (N = 103, see “Methods” and Supplementary Fig. 1). The resulting WM brain map included lateral and medial frontal regions, and also parietal, motor, and visual areas (Fig. 2A). The strongest n-back effect was located in medial frontal cortex (x,y,z = − 4,6,54; Z > 8.126, p_FWE-corr_ < 0.01), including a peak in ACC (x,y,z = − 6,20,46; Z > 8.126, p_FWE-corr_ < 0.01). Strong effects were also seen in left lateral frontal cortex, including a posterior lateral frontal peak (x,y,z = − 52,4,44; Z > 8.126, p_FWE-corr_ < 0.01) and a peak in the DLPFC (x,y,z = − 52,24,32; Z > 8.126, p_FWE-corr_ < 0.01).

To test the hypothesis that individuals with stable WM performance maintain PFC activity over time, we analyzed group by time interactions in each of these 4 frontal peaks (Fig. 2B–E). Significant group by time interactions were observed in medial frontal cortex [Fig. 2B, F(1,390) = 11.7, p < 0.001], ACC [Fig. 2C, F(1, 390) = 18.6, p < 0.001], lateral frontal cortex [Fig. 2D, F(1, 390) = 12.1, p < 0.001], and DLPFC [Fig. 2E, F(1, 390) = 17.3, p < 0.001]. In lateral frontal cortex (Fig. 2D,E), the wave-1 activity levels for decliners were actually higher than for the stable persons, and the difference was significant for 1-back [t(78) = 2.03 and 1.99, p’s ≤ 0.05 in D and E, respectively].

### Markers of general brain maintenance

#### Structural brain integrity

**Figure 3:**
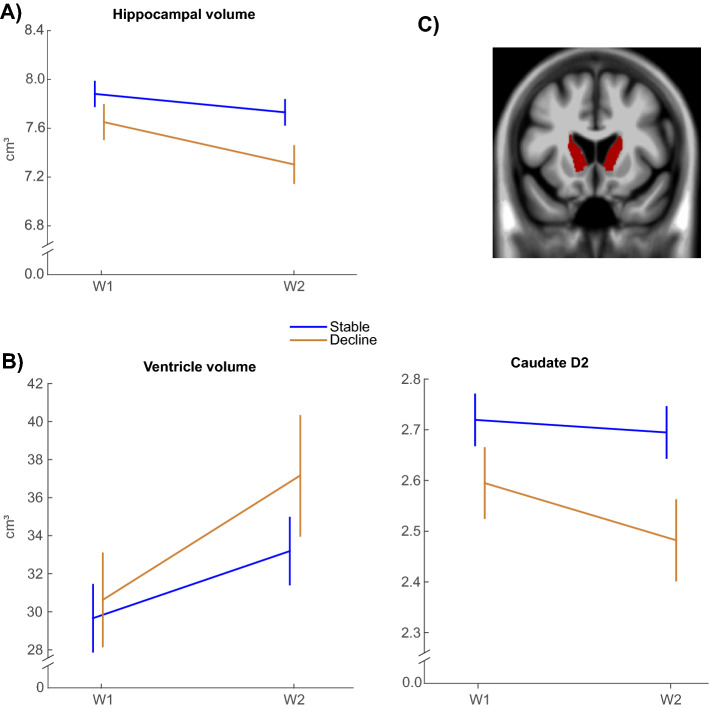
Comparison of stable and declining WM groups on markers of neuronal integrity and neurotransmission. (**A**) Hippocampal volume, (**B**) Ventricle volume, and (**C**) Caudate dopamine D2 binding potential. (Mean ± 1SE). W = Wave.

The analysis of hippocampal volume (Fig. 3A) revealed a significant main effect of time [F(1,78) = 61.0, p < 0.001], a trend-level main effect of group [F(1,78) = 3.6, p = 0.062], and a significant group by time interaction [F(1,78) = 9.5, p = 0.003]. Similarly, the analysis of ventricular volume (Fig. 3B) revealed greater enlargement in the declining group (group by time interaction [F(1,76) = 7.9, p = 0.006]), along with a significant main effect of time [F(1,76) = 196.1, p < 0.001] but not of group [F(1,76) = 0.8, p = 0.360].

#### Caudate dopamine D2-receptor availability

The analyses of caudate D2-receptor binding (Fig. 3C) revealed a significant main effect of group [F(1,75) = 4.5, p = 0.038], a borderline main effect of time [F(1,75) = 3.8, p = 0.054], and a non-significant group by time interaction [F(1,75) = 0.9, p = 0.345].

#### Vascular integrity

**Figure 4:**
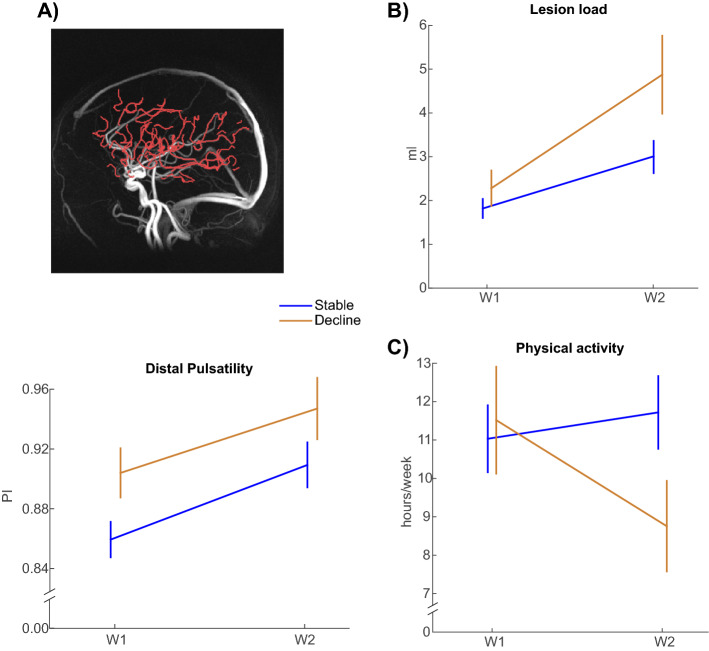
Comparison of Stable and Declining groups on markers of vascular health and self-reported physical activity. (**A**) Pulsatility in distal cerebral arteries. (**B**) White-matter lesions, and (**C**) Physical activity. (Mean ± 1SE). PI = Pulsatility index (unitless, see “Methods”); W = Wave.

Pulsatility in distal cerebral arteries (Fig. 4A) increased over time [F(1,67) = 15.0, p < 0.001]. A significant main effect of group was found [F(1,67) = 4.8, p = 0.032] but no group by time interaction [F(1,67) = 0.0, p = 0.842], reflecting higher pulsatility in the WM-declining group at both test waves. White-matter lesion load Fig. 4B) increased from baseline to follow-up [F(1,74) = 56.5, p < 0.001]. There was no main effect of group [F(1,74) = 2.7, p = 0.103], but the declining group showed elevated lesion progression over time [group by time interaction: F(1,74) = 4.0, p = 0.049].

### Physical activity

The analysis of self-reported physical activity (hours/week; Fig. 4C) revealed similar levels of activity at baseline across groups, whereas activity levels were reduced in the declining group at follow-up [group by time interaction: F(1,77) = 6.4, p = 0.014]. The main effects of time [F(1,77) = 1.5, p = 0.219] and group [F(1,77) = 0.6, p = 0.432] were non-significant.

### Supplementary results

First, the declining group was slightly and less educated than the stable group (see above), which may have contributed to higher levels (main effects) for crystallized and fluid ability, caudate dopamine, and pulsatility in the WM stable group. We therefore supplemented the ANOVA-based comparisons with ANCOVAs (with age and education as covariates). The pattern of results was highly similar, with the only difference being no group difference on vocabulary in the ANCOVA (Supplementary Table S1).

Second, we conducted supplementary ANOVAs of the larger sample with 77 WM stable and 41 WM declining individuals (N = 118, see Supplementary Fig. S1). The results were highly consistent with the findings from the main analyses. However, even though 118 individuals were included, compared to 80 in the main analyses, some effects that were significant in the main analyses did not reach significance in the supplementary analysis (Supplementary Table S2). This suggests that the less strict definition of WM stability/decline in these supplementary analyses constituted a weaker basis for the assessment of group differences in general brain maintenance.

Finally, change in 3-back WM (aggregated across the in-scanner and offline 3-back tasks) was correlated with longitudinal changes on the imaging markers (structural integrity, D2, lesions, pulsatility). In both the main (N_max_ = 80) and supplementary (N_max_ = 118) samples, WM change was significantly correlated with hippocampus (r_main_ = 0.26; r_supp_ = 0.22, p’s = 0.02) and ventricular (r_main_ = − 0.28; r_supp_ = − 0.24, p’s = 0.01) volume change. Corresponding brain-cognition longitudinal change-change correlations were non-significant for dopamine D2, lesion load, and pulsatility. For the measure of exercise, a borderline significant change-change correlation with WM was observed (r_main_ = 0.21, p = 0.06; r_supp_ = 0.19, p = 0.05).

## Discussion

Converging longitudinal data from in-scanner and offline versions of a 3-back task classified older participants into having stable or declining WM. The WM decline group was conservatively restricted to participants with above-chance 3-back performance at the first fMRI session, which made the stable and declining individuals more comparable in terms of baseline levels. Still, particularly on the offline 3-back task but also on the in-scanner 1- and 2-back tasks, the performance tended to be higher in the stable group than in the declining group already at baseline. Thus, to some degree, the WM classification reflected baseline differences that were magnified over time^21^.

The longitudinal fMRI analyses showed that individuals with stable WM maintained levels of activity in PFC regions, whereas declining WM related to reduced PFC activity over time. The finding that preserved WM in aging is linked to stable PFC activity is consistent with previous imaging studies^7^, and with pharmacological intervention studies showing that molecular manipulations can at least partially restore PFC neuronal WM firing in aged animals to youthful levels^22^, or conversely temporarily transform WM performance and PFC responses in young individuals such that they mimic those in older individuals^23^. The fMRI analyses also revealed higher baseline frontal activity during 1-back in the declining than in the stable group, which may reflect compensation for lower baseline WM capacity^14,15^.

After having established a relation between WM stability or decline with brain activity in frontal regions, we next explored *general* cognitive and brain maintenance (main effects of group and group by time interactions). Consistent with recent behavioral analyses^8^, we found that the stable WM group had superior performance at both waves on tests of fluid and crystallized abilities. Generality of brain maintenance was examined in several domains. Dopamine D2 measures in the caudate revealed significant differences between WM groups at both waves, and similar observations have been made in prefrontal regions^24^. Vascular health has been linked to maintained brain integrity and preserved cognition^25,26^, and a growing literature suggests that excessive microvascular pulsatility can have harmful cerebral and cognitive effects^19,27–29^, with marked influences on hippocampal structural and functional integrity and higher white-matter lesion burden^30–32^, which in turn can influence dopaminergic neurotransmission^12,33^. Moreover, excessive pulsatility may affect the blood–brain barrier^34^ and impair neurovascular coupling^35,36^, thereby influencing brain activity on tasks that require sustained processing over time^26^. Here, the stable WM group had less accumulation of lesions and lower levels of cerebral arterial pulsatility at both waves. High arterial pulsatility may reflect central arterial stiffness and elevated cerebrovascular resistance and indicates excessive exposure of the microvasculature to pulsatile stress. It should be noted that the stable WM group was slightly younger and had higher educational attainment than the WM-decline group. Level has been found to be influenced by education for both brain^37^ and cognition^38^ measures, but, except for crystallized ability all group differences remained significant in the supplementary analyses that controlled for age and education.

Significant interaction effects were observed for structural brain integrity, with less hippocampal atrophy and ventricular enlargement in the stable WM group. The results from the supplementary ANOVAs based on the larger sample and the continuous analyses of change-change associations provided additional support that WM change was related to both measures of atrophy. In addition, individuals in the stable WM group reported unchanging levels of weekly physical activity over 5 years along with a marked longitudinal reduction in the declining group, and the change-change analyses also suggested a relation between exercise and WM. These findings might reflect protective neurocognitive effects of physical activity, but on basis of these longitudinal observational data we cannot conclusively claim that a longitudinal reduction in weekly physical activity contributed to declining WM.

In summary, consistent with previous theoretical and empirical work, variability in longitudinal WM trajectories was strongly related to PFC functional responses, and in the analyses of general maintenance to rate of change on measures of neuronal integrity. A role of hippocampal atrophy in accounting for WM heterogeneity extends previous observations for episodic memory^3–5^. The hippocampus has been suggested to play a critical role also in WM^39^, but the finding of a similar pattern for the global (ventricle) measure is consistent with a more general contribution. Thus, the findings for neural integrity together with the observed level differences between stable and declining WM groups on markers of cognition, neurotransmission, and vascular health, support a contribution of general brain maintenance to well-preserved WM in aging.

## Methods

### Ethical approval and informed consent

The COBRA study was approved by the Regional Ethical board and the local Radiation Safety Committee of Umeå University, Sweden (2012-57-31M) and carried out in accordance with the Declaration of Helsinki. Written informed consent was obtained from all participants prior to testing.

### Participants: defining WM stability and decline

Figure S1 presents an overview of the flow of participants with 181 individuals at baseline (age range = 64–68 years), and 129 at the follow-up session. The participants were generally in good cognitive and physical health^12^. Five had missing/incomplete fMRI data, and 21 were excluded from the main analyses due to below-chance performance on the 3-back scanner task at the baseline session (M = 24.1, chance level = 31.5). This exclusion was done to ensure that the longitudinal classification of WM decline was not dominated by individuals with poor WM already at baseline. Moreover, interpretation of fMRI patterns would be unclear for individuals with below-chance performance. Assessment of longitudinal in-scanner 3-back data for the remaining 103 individuals classified 68 individuals as stable and 35 as having decline in WM (their longitudinal drop on the in-scanner 3-back task was less or more than 1 SD of the grand mean, M = 45.9, SD = 6.1, i.e., a drop of 7 or more defined decline). The offline 3-back performance pattern converged with the online pattern for the majority (80%) of individuals (3 individuals lacked offline follow-up data). It diverged for 12 individuals in the stable group (showing marked longitudinal reduction on the offline task) and 8 in the decline group (showing stable offline 3-back performance). Divergent cases were excluded from the main analyses of general brain maintenance, resulting in a final sample in the main analysis of 55 stable and 25 with WM decline.

In supplementary analyses of general brain maintenance (N = 118), we used a less stringent classification of WM stability or decline, which involved the 103 individuals who were classified on basis of the in-scanner 3-back task only, and an additional 15 of the 21 participants with chance performance on the in-scanner 3-back task (see Fig. S1). Based on their baseline- and follow-up performance on the offline 3-back task, the latter 15 could be classified into stable (N = 9) or declining (N = 6) WM (the remaining 6 had poor performance on the offline 3-back task at both sessions and were excluded).

### Offline tasks

Data from a columnized numerical 3-back task from the COBRA offline battery^11^ was analyzed together with the in-scanner 3-back task to classify participants as stable or declining. This offline 3-back task is similar to the in-scanner version and has high reliability (max score = 108, reliability = 0.90). The offline test battery included one measure of crystallized ability (a 30-item synonym test). For fluid ability, three speed-of-processing measures were included (letter-, number-, and figure comparison) and formed a composite score (i.e., the numbers of correct responses on each task were summed).

### MRI and PET markers of general brain maintenance

#### Structural brain integrity

MRI was performed on the same 3 T Discovery MR 750 scanner (General Electric, WI, US). Anatomical T1-weighted images were acquired with a 3D fast-spoiled echo sequence, collected as 176 slices with a thickness of 1 mm. TR = 8.2 ms, flip angle = 12 degrees, and field of view = 25 × 25 cm. The analysis was masked for regions in grey and white matter only. FreeSurfer (v. 6.0) segmentations were performed with the three-step longitudinal pipeline^40^. Bilateral hippocampal and (left + right lateral) ventricular volumes were used as measures of regional and global structural brain integrity.

#### Dopamine neurotransmission

For the PET measure of dopamine D2 receptor availability, a 55-min, 18-frame dynamic PET scan was acquired during rest following intravenous bolus injection of approximately 250 MBq ^11^C-raclopride (Discovery PET/CT 690 scanner, General Electric, WI, USA). The PET images were motion-corrected and co-registered with the structural T1-weighted MR images from the corresponding session (baseline and follow-up) using Statistical Parametric Mapping (SPM12). FreeSurfer segmentations and pre-processed PET data were used to estimate partial volume-corrected regional radioactivity concentrations using the symmetric geometric-transfer matrix implemented in FreeSurfer^41^ per time frame. The size of the correction kernel was estimated empirically (point-spread function (PSF) of 2.5 mm; isotropic), to achieve a similar level of correction as in prior work^41^. Binding potential (BP_ND_) was calculated with multilinear reference-tissue model (MRTM) on dynamic partial volume-corrected data, with cerebellar GM radioactivity as reference. We focused on the caudate, which has a high density of D2 receptors^42^, and elevated caudate D2 release was observed in a PET study of numerical 3-back in comparison with 1-back^43^.

#### Cerebrovascular integrity

For vascular health, white-matter hyperintensities were segmented from a FLAIR image (48 slices, slice thickness = 3 mm, TE = 120 ms, TR = 8000 ms, field of view = 24 × 24 cm) using the lesion-growth algorithm^44^, as implemented in the LST toolbox (version 2.0.14 for SPM12). The algorithm segmented the T1-weighted images into cerebrospinal fluid, grey matter, and white matter, and this information was combined with the co-registered FLAIR intensities to calculate lesion-belief maps. The maps were thresholded (κ = 0.3, defined by visual inspection) to obtain an initial binary lesion map, which was grown along hyperintense neighbouring voxels in the FLAIR image, and thresholded at 50% to yield a binary lesion map. From this, total volume (ml) and number of lesions were obtained.

4D flow MRI data for cerebral arterial pulsatility were collected with a PC-VIPR (Phase Contrast Vastly undersampled Isotropic-voxel Projection Reconstruction) sequence that provides time-resolved vascular flow velocities in all spatial directions, time-resolved over the cardiac cycle, and with whole-brain coverage^45,46^. Imaging parameters: 5-point velocity encoding (venc): 110 cm/s, TR/TE: 6.5/2.7 ms, flip angle: 8$^\circ$, number of radial projections: 16,000, reconstructed temporal resolution: 20 frames per cardiac cycle, matrix size at acquisition: 300 × 300 × 300, imaging volume: 22 × 22 × 22 cm^3^, matrix size after reconstruction: 320 × 320 × 320, spatial resolution: 0.69 mm isotropic. Scan time was approximately 9 min.

Velocity data (x, y, z, t) and a complex-difference (CD) angiogram used to highlight vascular structure were reconstructed from the 4D flow scans. A vascular centerline representation was obtained from the CD volume, and pulsatile flow waveforms were sampled automatically along all centerline branches^47^. Small, distal cerebral arteries (Fig. 4A) were first automatically identified using a diameter threshold (< 1.25 mm) and waveform information to separate arteries and veins, with manual pruning to assure no venous contamination. The final distal arterial waveform was obtained by averaging flow waveforms from all identified distal cerebral arteries^48^, and the pulsatility index (a unitless measure defined as the waveform amplitude divided by mean flow rate over the cardiac cycle) was used to characterize the waveform.

### Physical activity

Weekly self-reported physical activity was computed from the participants’ responses to an activity questionnaire. In keeping with previous COBRA studies^49^ we focused on activities that are physically demanding and that individuals are actively engaged in: walking, bicycling, jogging, strength training, and other sports such as tennis and golf (*each* of these activities were performed by > 20% of the participants at least once a week, with some being engaged in many activities per week and others in only one or a few).

### Functional MRI during n-back

The n-back fMRI task^13^ was administered in the MRI scanner (a 3 Tesla Discovery MR 750 scanner, General Electric, WI, US) in an identical manner during both test waves. The n-back performance data (sum of correct yes/no responses) were obtained from a numerical n-back task. A sequence of single numbers appeared on the screen, and each number was shown for 1.5 s, with an ISI of 0.5 s. For each item, participants reported if the number currently seen on the screen was the same as the number shown 1, 2, or 3 digits back. Participants responded by pressing one of two adjacent buttons with their right-hand index or middle finger indicating “yes” or “no”. A single fMRI run with 9 blocks for each condition (1-, 2-, and 3-back) was performed in random order (inter-block interval: 22 s), where each block consisted of 10 items with 4 targets. The trial sequence was the same for all participants with two lures (a single 2-back lure within two of the 3-back blocks). The n-back blocks were counterbalanced. A header preceded each subtest and indicated the actual n-back condition to be performed.

BOLD-contrast sensitive scans were acquired using a T2*-weighted single-shot gradient echoplanar-imaging sequence. The parameters were: 37 transaxial slices, 3.4 mm thickness, 0.5 mm spacing, TE/TR = 30/2000 ms, 80° flip angle, 25 × 25 cm field of view, and a 96 × 96 acquisition matrix (Y direction phase encoding). A total of 330 volumes were collected. At the onset, 10 dummy scans were collected and discarded. Preprocessing of the fMRI data included slice-timing correction to the first slice of each volume and motion correction by realignment and non-linear unwarping. The realignment routine calculates 3 translation parameters (x, y, and z) and 3 rotation parameters (roll, pitch, and jaw) reflecting the location of each volume compared to the first volume. If a movement of more than 2 mm from volume to another was detected, Artrepair^50^ was used to interpolate over that volume. 9 out of 160 fMRI-runs used in BOLD-change-plots were mended with Artrepair^50^. Each fMRI-series were coregistered to the T1-image for the corresponding subject and wave. The T1-images were segmented into grey matter, white matter, and CSF-probability maps, which were used to create subject-specific templates, which then were used to create a sample-specific template using DARTEL, resulting in flow-field files. By flow-field files, the fMRI volumes were transformed first to subject-template-space and then to sample-dartel-template space and then affine aligned to MNI standard space, and spatially smoothed by convolving with an 8-mm FWHM Gaussian kernel. As a first-order analysis, a general linear model with regressors for each load condition (1-back, 2-back, 3-back), convolved with the canonical hemodynamic response function, the 6 realignment parameters from the movement correction as covariates of no interest, and a constant term was set up. Each block was 20 s and there were 9 blocks per n-back-condition. Three rest-fixation periods of 20 s each defined an implicit baseline. Before regression the BOLD-signal was high-pass filtered with a cut-off at 130 s. The regression resulted in one slope-file (beta-file) for each regressor and fMRI-run. A contrast with summed slope-files for the three back-regressors was set up for each subject at first wave and used in a group t-test, N = 103. BOLD-change in % was calculated for several brain coordinates of interest by slope divided by the constant and multiplicated by 100. Processing and analyses of fMRI-data was done with SPM12, and simplified batching and inspection of result with in-house program, *DataZ*.

### Statistical analyses

Repeated-measures ANOVA models tested for main effects of group, time, and group by time interactions for longitudinal fMRI-signal changes and markers of general brain maintenance. Change-change analyses assessed correlations between (i) the difference between the two waves of 3-back-WM (in-scanner and offline results summed for each subject) and (ii) differences between the two waves on the variables of interest. T-and χ^2^ tests were used to compare the groups on background variables. Analyses were run in SPSS (version 27) and Matlab (version R2014B). Due to technical issues or being identified as statistical outliers^51^ some participants were excluded from specific analyses. The exact numbers (degrees of freedom) are given for each analysis.

## Supplementary Information

Supplementary Information.

## Data Availability

The datasets generated and analysed during the current study are available from the corresponding author on reasonable request.
