# Supplementary material for: Longitudinal stability in working memory and frontal activity in relation to general brain maintenance
Source: Sci Rep. 2022 Dec 5;12:20957. doi: 10.1038/s41598-022-25503-9 (PMC9722656; doi:10.1038/s41598-022-25503-9)
Supplement: Supplementary file 1 — Supplementary Information. [file 41598_2022_25503_MOESM1_ESM.pdf]

Longitudinal stability in working memory and frontal activity in relation to general brain maintenance

Lars Nyberg, Nina Karalija, Goran Papenberg, Alireza Salami, Micael Andersson, Robin Pedersen, Tomas Vikner, Douglas D. Garrett, Katrine Riklund, Anders Wåhlin, Martin Lövdén, Ulman Lindenberger, & Lars Bäckman

- Supplementary Figure S1
- Supplementary Table S1
- Supplementary Table S2

S1

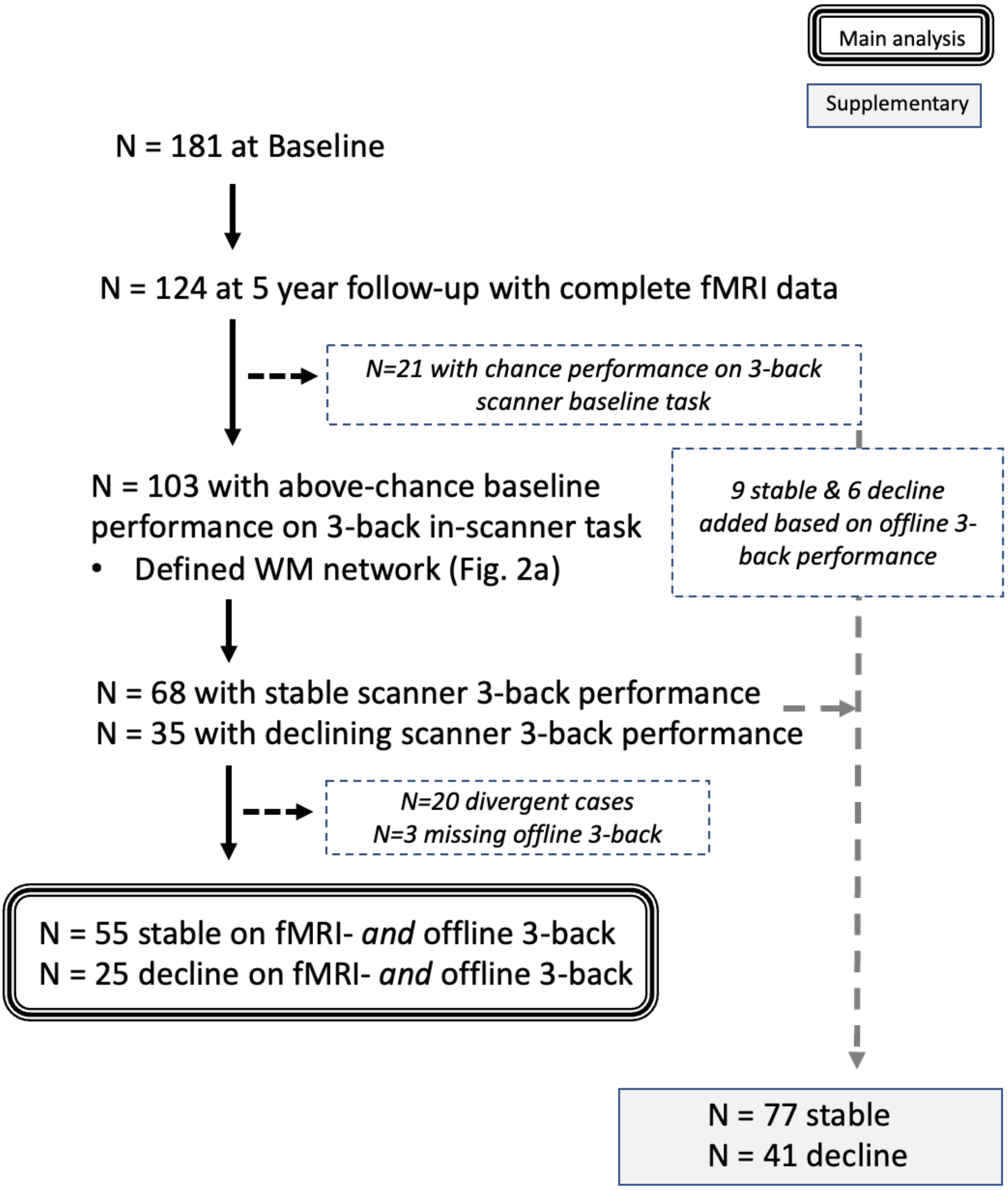

Figure S1: Flow of participants to define WM brain network and classification of stability or decline in longitudinal WM that was used in main and supplementary analyses of general brain maintenance.

Supplementary Table S1: *Outcomes from supplementary ANCOVAs with age and education as covariates*

	Group	Time	Interaction	df
Speed	0.002	0.931	0.748	73
Vocabulary	0.182	0.784	0.189	74
CaudD2	0.044	0.065	0.439	73
DistalPI	0.042	<0.001	0.739	65

Note: CaudD2 = caudate Dopamine D2; PI = Pulsatility Index, df=degrees of freedom

Supplementary Table S2: *Significance values from main and supplementary ANOVAs*

Main	Nmax=80, p-values			
	Group	Time	Interaction	df
Speed	0.006	0.892	0.631	75
Vocabulary	0.015	0.890	0.270	76
Excercise	0.432	0.219	0.014	77
Lesion	0.103	0	0.049	74
Ventr.volume	0.360	0	0.006	76
HCvol	0.062	0	0.003	78
CaudD2	0.038	0.054	0.345	75
DistalPI	0.032	<0.001	0.842	67

Supplementary	Nmax=118, p-values			
	Group	Time	Interaction	Df
Speed	0.015	0.631	0.365	113
Vocabulary	0.008	0.907	0.558	112
Excercise	0.871	0.080	0.027	114
Lesion	0.284	0	0.128	106
Ventr.volume	0.876	0	0.073	112
HCvol	0.088	0	0.004	114
CaudD2	0.154	0.045	0.409	113
DistalPI	0.022	<0.001	0.452	98

Note: Ventr. = ventricular; HCvol = Hippocampus volume; caudD2 = caudate Dopamine D2; PI = Pulsatility Index, df=degrees of freedom.
